# Amal Saif Al-Maani: tackling drug-resistant pathogens

**DOI:** 10.2471/BLT.21.030821

**Published:** 2021-08-01

## Abstract

Amal Saif Al-Maani talks to Andréia Azevedo Soares about antimicrobial resistance and the need for multisectoral approaches to mitigating its development.

*Q: What inspired your interest in the field of antimicrobial resistance?*

A: After I got my medical degree, I went to Canada for a paediatric residency programme at The Hospital for Sick Children in Toronto and developed a particular interest in paediatric infectious diseases and infection control. I was fortunate enough to obtain a fellowship in 2010 and it was around that time that I started to really get interested in antimicrobial resistance. I remember a very sick newborn girl being admitted to the intensive care unit with a vancomycin-resistant enterococcus (VRE) infection in her bloodstream. My job as an infection control fellow at that time was to make sure the VRE didn’t spread to other patients and to find out how and where the girl had become infected. Our investigations revealed significant transmission of VRE in the community. The newborn’s mother had been infected by the baby’s grandmother during a visit to the family home at Christmas. At the time it seemed remarkable to me, partly because of the banality of the likely transmission event – probably the grandmother failing to wash her hands properly and passing this life-threatening pathogen to her pregnant daughter and then to the newborn baby. Fortunately, after two months of hospital care, the baby was saved and there was no transmission to other babies in the unit.

The following year, I started to practise as a consultant in infectious diseases at the Royal Hospital in Oman. The first case I was called to see was a child who had just had cardiac surgery. The surgery had been successful, but the child had been infected with methicillin-resistant *Staphylococcus aureus* (MRSA), another multi-drug-resistant pathogen. She’d had to go back to the operation room to have some infected tissue removed and needed an effective antibiotic. We treated her with vancomycin, an antibiotic that can have serious side-effects including nephrotoxicity (damage to kidneys) and ototoxicity (damage to hearing). Fortunately, the girl recovered. I thought at the time what would have happened if we hadn’t been able to treat her? And what is going to happen when there is a bug we cannot treat? In the past ten years we have seen more and more drug-resistant cases and things are getting complicated.

“We have reached a point where urinary tract infections cannot be treated in the community.”

*Q: Which pathogens cause you the greatest concern?*

A: I am most concerned by new broad-spectrum antibiotic-resistant bacteria, including VRE*,* and the carbapenem-resistant Enterobacterales. Their treatment is becoming increasingly challenging, invasive infections are often fatal, and preventing their transmission is a nightmare. Regarding fungi, *Candida auris* worries us most. It is resistant to the antifungal drugs commonly used to treat *Candida* infections and we have started to see reports from some countries about strains that are resistant to all three classes of antifungals. There have been a number of outbreaks of *C. auris* in health-care settings which makes it a matter of particular concern, and to make matters worse it can easily be misidentified in labs. Misidentification then leads to delay in management including prevention of further transmission. So, we really need to work on preventive strategies for antimicrobial resistance supported by effective timely surveillance as part of the broader antimicrobial resistance response. Otherwise modern medicine itself, and in particular the surgical interventions we have become accustomed to, may become a thing of the past.

*Q: You are credited with establishing a system for antimicrobial resistance surveillance in Oman. What was the starting point for that effort?*

A: We wanted to design a system that captured both the pathogens and the carriers of the pathogen in their context. We did this by gathering data in selected country hospitals which had well defined catchment areas and thus gave us a good idea of the prevalence and incidence of infection within the different communities. We also captured data on antimicrobial use, allowing us to monitor use by region, hospital and health centre. Using these data sets we were able to identify links between emerging resistance and antimicrobial use and consumption. Importantly, we use the data to provide feedback to health professionals working in the public and private sectors so that they can see how they are doing in each period. We also provide guidance to clinicians regarding emerging resistance – for example which antibiotic is increasingly ineffective against which specific pathogen.

*Q: How do you capture data?*

A: Via the electronic medical and laboratory record system and using the reference hospitals. Essentially, we take a reference hospital for each province, which is generally the largest hospital and is equipped with the laboratory and staff required to produce quality data. The data are collated in a national database that we also established. Patient data are segregated by sex, age group, pathogen and treatment. We also monitor whether the infection is hospital or community acquired. Our aim is to capture representative data for the entirety of the country.

*Q: You have been recognized for your work in promoting a multisectoral approach to tackling the antimicrobial resistance challenge. Why is this important?*

*A: *Because antimicrobial resistance is a multisectoral problem, a fact that is brought home to me every day with the patients I see. Each infection raises multiple questions, starting with where did the patient get it? What was the route of transmission? Did it happen here in the hospital, at home? From misuse or overuse of antibiotics? Nobody can get antibiotics over the counter in Oman; you have to go to the pharmacy with a physician’s prescription and both prescription and dispensing is monitored and supervised by the Ministry of Health, whether in the public or private sector. However, that does not prevent people from misusing drugs or from bringing antibiotics in from less regulated countries. Then there is transmission through the food people consume – perhaps chicken in which antibiotics were used as a growth promoter or for prophylactic purposes. In some instances, the transmission route can be quite tenuous, for example where wastewater containing human and/or animal excreta carrying antimicrobial-resistant pathogens is used to water the plants that animals eat. We believe that our sewage systems may contain a lot of antimicrobials and because of that pathogens may be evolving in the environment. In order to confirm that hypothesis we need to start doing surveillance in the sewers too. So, following these different transmission paths you realize that antimicrobial resistance is not just a health sector problem, it needs to be addressed in connection with other sectors, which means people in other sectors have to be made aware. That is why we promote integrated approaches to human, animal and environmental health, a One Health approach.

“We need a One Health approach implemented across all sectors and communities.”

*Q: How did you go about this in Oman?*

A: We started by running an awareness campaign during the second half of 2016 which was designed to elicit multisectoral support across a range of institutions, including the ministries of agriculture and information, the universities and local councils. An important part of the campaign was making people face the stark reality of the situation we are in, so we shared stories from the front line of health service delivery. We wanted to let people know that we have reached a point where, for example, urinary tract infections cannot be treated in the community because the simple antibiotics that used to be taken orally outside are no longer effective. Patients need intravenous antibiotics.

*Q: What impact did that campaign have?*

A: It is hard to say and there have been no formal impact studies. But one outcome was the increased involvement of the Ministry of Information which embraced the issue and gave its support to the campaign. This has been crucial in getting the attention of national media outlets who have helped get the word out but have also helped present information in the most effective way. Journalists started to write about the problem in simple language –about the need for proper hand hygiene, rational use of antibiotics and other preventive strategies. That has really helped drive our community campaigns. We have also focused efforts on engaging with communities as partners, sharing with them what is being done and welcoming their input.

*Q: How has the pandemic impacted efforts to tackle antimicrobial resistance in the region?*

A: Well, on the one hand it has drawn attention away from antimicrobial resistance but it is also helping in some ways. Hand washing is a good example. The pandemic has put a spotlight on the importance of hand hygiene, but it has also made people more aware of the microbiological risks to which they are exposed more generally. In many ways, improvements in living standards have made people less aware of those risks and have caused them to lower their guard. Hand hygiene is the prime example of this. Ninety-nine times out of a hundred our contact with pathogenic microbes is mediated through our hands. COVID-19 has made us aware of this and it has changed our behaviours. But that behaviour change needs to be sustained, so we need to keep talking about it.

*Q: As an infection control expert in the midst of a pandemic, what keeps you awake at night?*

A: The next wave of COVID-19 keeps me awake. Or the wave after that. I worry about floods of patients overwhelming hospitals. I also worry about infection prevention and control strategies not being maintained, especially as more people get vaccinated and there is the impression that we are coming to the end of the pandemic. With low levels of vaccination at the global level, there is a very real chance that this virus will be circulating for several years. So, discipline must be maintained. But I’m also optimistic, not least because of recent developments in biotechnology. There is no reason we cannot apply the platform technologies that brought us vaccines to the development of new drugs and vaccines to support the antimicrobial resistance response. But we also need to look beyond the laboratory and make sure that our efforts are more comprehensive and organized. We need a One Health approach implemented across all sectors and communities. We really have no choice.

